# High rate of macrolide resistance and closely genetically related *Mycobacterium abscessus* complex strains identified among both cystic fibrosis and non-cystic fibrosis patients within two countries

**DOI:** 10.1128/spectrum.01056-24

**Published:** 2024-10-23

**Authors:** Matúš Dohál, Věra Dvořáková, Michaela Hromádková, Martina Pinková, Jana Amlerová, Marek Schwarz, Andrea Spitaleri, Federico di Marco, Jarmila Hnilicová, Eduard Gondáš, Michael E. Rasmussen, Igor Porvazník, Ivan Solovič, Daniela M. Cirillo, Juraj Mokrý

**Affiliations:** 1 Biomedical Centre Martin, Jessenius Faculty of Medicine in Martin, Comenius University, Bratislava, Slovakia; 2 National Institute of Public Health, Prague, Czechia; 3 Charles University, Faculty of Medicine in Pilsen, Faculty Hospital, Pilsen, Czechia; 4 Institute of Microbiology of the Czech Academy of Sciences, Prague, Czechia; 5 Division of Immunology, Transplantation and Infectious Diseases, Emerging Bacterial Pathogens Unit, IRCCS San Raffaele Scientific Institute, Milan, Italy; 6 Vita-Salute San Raffaele University, Milan, Italy; 7 Department of Genetics and Microbiology, Faculty of Science, Charles University, Prague, Czechia; 8 Department of Pharmacology, Jessenius Faculty of Medicine in Martin, Comenius University, Bratislava, Slovakia; 9 International Reference Laboratory of Mycobacteriology, Statens Serum Institut, Copenhagen, Denmark; 10 National Institute of Tuberculosis, Lung Diseases and Thoracic Surgery, Vyšné Hágy, Slovakia; 11 Faculty of Health, Catholic University, Ružomberok, Slovakia; Beijing Institute of Genomics, Chinese Academy of Sciences, Beijing, China

**Keywords:** *M. abscessus*, transmission, epidemiology, DCC, resistance, non-tuberculous mycobacteria

## Abstract

*Mycobacterium abscessus* is an emerging opportunistic pathogen affecting patients with chronic lung diseases, primarily cystic fibrosis (CF), or those under immunosuppression. Hence, investigations into the epidemiology and transmission of *M. abscessus* and accurate antibiotic susceptibility data are essential for the effective treatment of infections caused by this pathogen. This retrospective nationwide study included all clinical *M. abscessus* isolates (*n* = 59) from 29 patients diagnosed in the Czech Republic and Slovakia between 2018 and 2023. Whole genome sequencing (WGS) was performed to identify clusters and classify isolates into predominant circulating clones (DCC). Subspecies identification of unique isolates showed subspecies *abscessus* as the most prevalent (69.0%). The results of drug-susceptibility testing showed that 65.5% of all isolates were resistant to at least three antibiotics tested. CF patients under 24 years of age were the most at-risk group for *M. abscessus* infection. WGS identified seven clusters (including two cross-border) comprising CF and non-CF patients with a total clustering rate of 48.3%. One cluster involved patients infected with subspecies *massiliense* strains differing by 0 single nucleotide polymorphisms hospitalized in the same center. Furthermore, we identified representatives of all major DCCs. This study revealed predominant *Mycobacterium abscessus* complex clones circulating in the Czech Republic and Slovakia. The results show the high discriminatory power of WGS in the molecular epidemiology of *M. abscessus* and provide supporting evidence of direct or indirect cross-transmission of subspecies *massiliense* among both CF and non-CF patients.

**IMPORTANCE:**

This study highlights the importance of understanding *Mycobacterium abscessus* transmission because it poses a growing threat to vulnerable populations, especially young cystic fibrosis patients. Investigating how it spreads and which antibiotics work best is crucial for effective treatment. This research used whole genome sequencing to track *M. abscessus* and found evidence of potential transmission between patients, including across borders. The findings suggest that dominant strains are circulating and some patients may be infected through direct or indirect contact. This knowledge can inform infection control and treatment strategies.

## INTRODUCTION

Non-tuberculous mycobacteria (NTM) are mainly environmental microorganisms that can lead to infections among susceptible populations. The frequency of pulmonary diseases caused by these bacteria has risen in recent decades (1). *Mycobacterium abscessus* complex (*MABC*) is the most prevalent and clinically relevant rapidly growing NTM (2). The *MABC*, including subspecies *abscessus* (*Mabs*), *massiliense* (*Mmas*), and *bolletii* (*Mbol*), is characterized by its capacity to induce severe pulmonary disease, which can be devastating for individuals with cystic fibrosis (CF) (3). A lung transplantation can substantially enhance the quality of life and increase the life expectancy of certain CF patients with advanced lung disease. However, the presence of the *MABC* before the transplantation may be a contraindication due to concerns for increased morbidity, mortality, and risk of post-transplant transmission (4). This complex is also characterized by a high incidence of mutational and acquired resistance to commonly used antibiotics, resulting in treatment success rates for *Mabs* pulmonary disease of only 25%–58% (5, 6). Nevertheless, a substantial percentage of *Mmas* clinical isolates is susceptible to macrolides like clarithromycin when evaluated through *in vitro* antimicrobial susceptibility. *Mmas* susceptibility is primarily attributed to a substantial deletion in the *erm*(41) gene (which encodes a methylase that methylates 23S ribosomal rRNA), rendering it nonfunctional and making the mycobacteria responsive to macrolides, leading to cure rates typically ranging from 80% to 90% (7). Resistance to macrolides is in all three subspecies encoded predominantly by mutations in *rrl* gene (23S rRNA), whereas mutations in the *rrs* gene (16S rRNA) are usually responsible for resistance to aminoglycosides (8, 9, 10, 11).

Although it was initially thought that *M. abscessus* was only acquired independently from the environment, recent studies have revealed that individuals with CF can also become infected through person-to-person transmission in the hospital, but it is still unclear if this transmission is direct or indirect via an environmental intermediate such as long-lived aerosols or fomite spreads (12–14). A comprehensive large-scale whole genome sequencing (WGS) analysis of global *M. abscessus* isolates from CF patients has revealed that a large proportion of CF patients is infected by one of three dominant circulating clones (DCCs) (15, 16). Furthermore, DCC representatives have demonstrated increased virulence *in vivo* and *in vitro* (17). While the results indicate the potential presence of DCCs in CF patients, only limited investigations have employed WGS with clinical and epidemiological data to explore if similar events occur in non-CF patients.

The main objective of the study was to clarify the possibility of cross-transmission of *MABC* among CF and non-CF patients by performing WGS analysis, investigating clinical-epidemiological data, and classifying *MABC* into DCCs. Another goal was to evaluate the drug resistance in all *MABC* clinical isolates collected from 2018 to 2023 in the Czech Republic and Slovakia. Additionally, we assessed the phenotypic drug susceptibility testing (pDST) results for particular drugs associated with mutations identified by the GenoType NTM-DR (Hain Lifescience GmbH, Nehren, Germany) and WGS.

## MATERIALS AND METHODS

### *MABC* isolates

A total of 59 *MABC* isolates recovered from 29 patients were referred to the National Reference Laboratory for Mycobacteria, National Institute of Public Health, Prague, Czech Republic, and National Reference Mycobacteriology Laboratory, National Institute for TB, Lung Diseases and Thoracic Surgery, Vyšné Hágy, Slovakia, between January 2018 and December 2023. All isolates were recovered from pulmonary specimens.

### pDST

In pDST, isolates were cultivated on Middlebrook 7H10 agar using the following antibiotic concentrations: 0.5 mg/L, 1.0 mg/L, 2.0 mg/L, 4.0 mg/L, 8.0 mg/L, 16.0 mg/L, 32.0 mg/L, and 64.0 mg/L of clarithromycin, azithromycin, amikacin, linezolid, ciprofloxacin, and moxifloxacin. Incubation was carried out at 37°C for a period ranging from 3 to 5 days for all antibiotics. In cases where no resistance to clarithromycin was observed during this interval, an extended incubation period of 14 days was employed to detect inducible resistance. The breakpoints for interpreting susceptibilities followed the Clinical and Laboratory Standards Institute guidelines (18).

### Genotypic drug susceptibility testing and subspecies identification

For genotypic drug susceptibility testing (gDST), DNA from all isolates (*n* = 59) was extracted using the Hain Genolyse Kit and underwent a PCR program according to the manufacturer’s instructions (Hain Lifescience, Nehren, Germany). The assay enables differentiation of three *MABC* subspecies (*Mabs*, *Mmas*, and *Mbol*) as well as the detection of inducible macrolide resistance [*erm*(41), C28T], inherent macrolide resistance (*rrl* gene, A2058C, A2058G, A2058T, A2059C, A2059G, and A2059T), and constitutional aminoglycoside resistance (*rrs* gene, A1408G, T1406A, and C1409T).

### WGS

WGS was performed on 31 *MABC* isolates originating from 23 patients (73.9% of all patients diagnosed with *MABC* between 2018 and 2022). Culture material for sequencing was not available for the remaining six patients, as these patients were only reported to the register without sending the culture material to the reference laboratory. *MABC* isolates were grown in Middlebrook 7H10 agar. DNA was extracted from 1 mL of heat-inactivated (95°C for 30 min in the biosafety level 3 laboratory) early-positive culture using the QIAamp DNAMini Kit (Qiagen, Hilden, Germany). DNA concentration was quantified by Qubit 4 technology using the Qubit dsDNA HS Assay Kit (Thermo FisherScientific, Waltham, USA) and normalized to 0.2 ng/µL to be used as input for the Nextera XT Library Preparation Kit (Illumina, San Diego, CA, USA). Library preparation was performed according to the manufacturer’s instructions. Libraries were batched and sequenced with an Illumina MiSeq (Illumina, California, USA) using 2 × 150 bp paired-end chemistry.

### Bioinformatics analysis

The Illumina data underwent processing via the MTBseq pipeline’s Nextflow implementation (Github: Allen13x/NF_TBSEQ) (19). The analysis utilized the *M. abscessus* reference genome ATCC 19977 (CIP 104536T). The construction of the transmission tree employed Grapetree v2.2, utilizing the Multiple Sequence Alignment file obtained from the pipeline’s Amend step (20). Genetic closeness was determined with a threshold of 25 single nucleotide polymorphisms (SNPs), and visualization was conducted through Cytoscape v3.9.1 aided by the RCy3 package v2.14 (21).

For comprehensive phylogenetic analysis, we integrated 79 public genomes from Ref. (17), representative of the three *MABC* dominant circulating clones. The phylogenetic tree generation utilized RAxML v8.2.12 software (PMID: 24451623), and the representation was facilitated using the iTOL web service (PMID: 33885785).

## RESULTS

### Data set characterization

**Fig 1:**
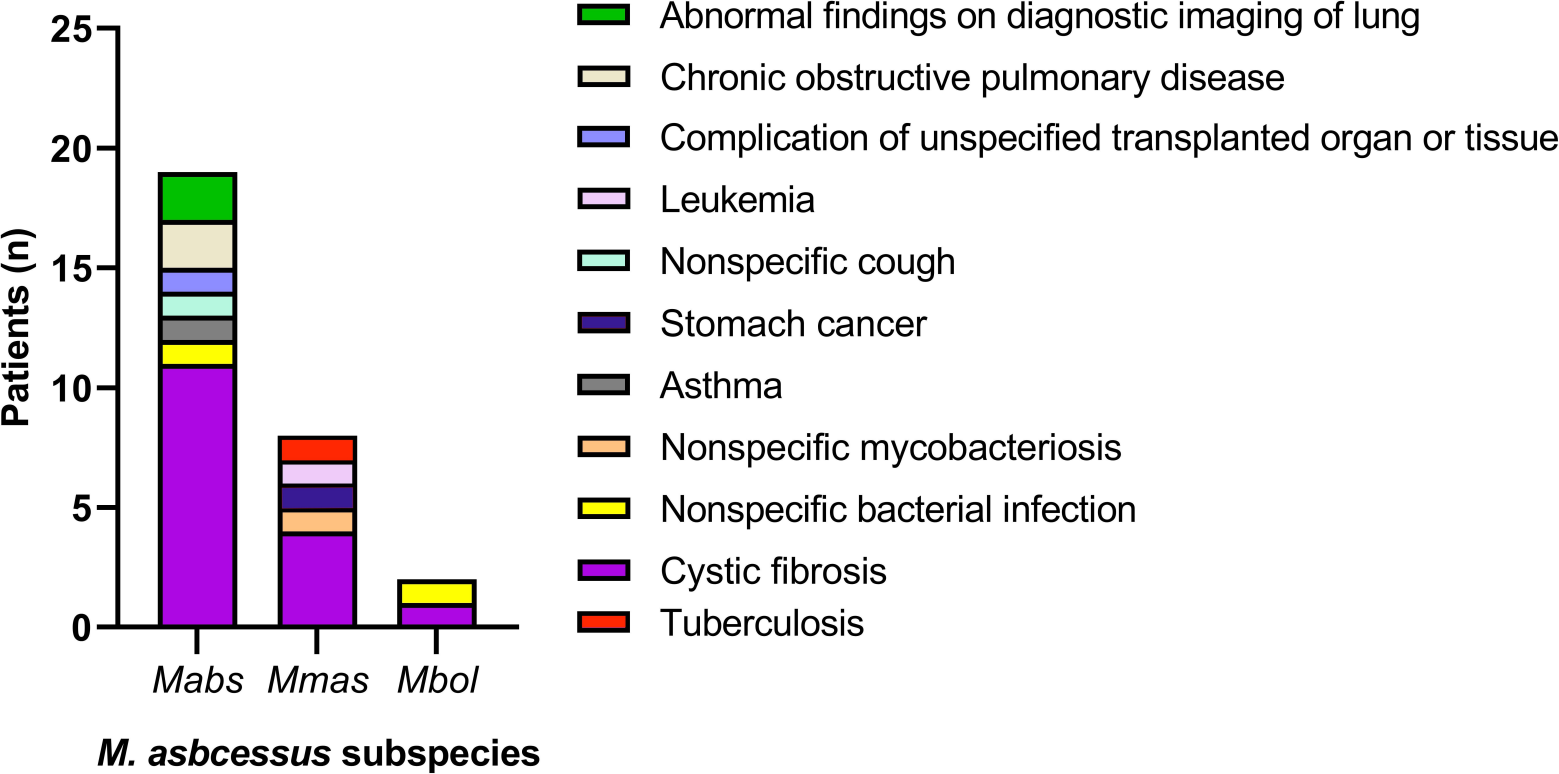
Primary diagnosis among patients enrolled in the study with *MABC. Mabs*, *M. abscessus* subsp. *abscessus; Mmas*, *M. abscessus* subsp. *massiliense*; and *Mbol*, *M. abscessus* subsp. *bolletti*.

The present study included 59 *MABC* isolates from 29 patients diagnosed in 2018–2023 (Table 1). CF was the most common comorbidity of patients (Table 1; Fig. 1). GenoType NTM-DR allowed the identification of 20 isolates as *Mabs*, 7 as *Mmas*, and 2 as *Mbol* (Table 1). One strain identified as *Mbol* was reclassified as *Mmas* based on WGS analysis. We identified no changes in subspecies distribution within paired isolates from the same patients. Moreover, no differences in the distribution of specific sublineages and no mixed *MABC* infections were detected between the CF and non-CF patient cohorts.

**TABLE 1:** Summarized characterization of the *Mycobacterium abscessus* complex isolate data set^*a*^

Characteristics	*N* (%) of patients
Age	
0–14	6 (20.7)
15–24	11 (37.9)
25–54	3 (10.3)
55–64	1 (3.4)
>65	8 (27.6)
Diagnosis	
CF	16 (55.2)
Non-CF	13 (45.8)
*M. abscessus* subspecies	
*Mabc*	20 (69.0)
*Mmas*	7 (24.1)
*Mbol*	2 (6.9)

^*a*^ *Mabs*, *M. abscessus* subsp. *abscessus*; *Mmas*, *M. abscessus* subsp. *massiliense*; and *Mbol*, *M. abscessus* subsp. *bolletii*.

### pDST and gDST

Results of pDST and genetic mutations encoding resistance to macrolides and aminoglycosides GenoType NTM-DR are summarized in Table 2 (results remained consistent across paired isolates from the same patient). Macrolide resistance was most commonly observed, with 19 isolates (65.5%) (12 *Mabs*, 5 *Mmas*, 2 *Mbol*) resistant to clarithromycin and 20 isolates (68.9%) resistant to azithromycin (13 *Mabs*, 5 *Mmas*, 2 *Mbol*). Twenty (69.0%) carried the *erm*(41)T28, and 14 demonstrated acquired resistance to clarithromycin. Despite expressing an *erm*(41)T28 gene and a wild-type *rrl* gene, three *Mabs* isolates exhibited clarithromycin sensitivity. Three *Mmas* isolates also exhibited *erm*(41)T28, but this gene of *Mmas* is nonfunctional, leading to macrolide sensitivity despite a developed *erm*(41)T28 band based on the manufacturer’s instructions GenoType NTM-DR (except for isolate 26620 with an additional *rrl* mutation). Additionally, mutations encoding resistance identified by WGS were in 100% concordance with GenoType NTM-DR except for two *Mmas* subspecies exhibiting the inducible resistance by line probe assay. Using pDST as the reference standard, GenoType NTM-DR and WGS identified macrolide resistance with a sensitivity of 94.4% [95% confidence interval (CI) 72.71–99.86]. However, the diagnostic specificity of these methods was lower at 72.73% (95% CI 39.03–93.98) (Table S1).

**TABLE 2:** Susceptibility patterns for clarithromycin, azithromycin, amikacin, linezolid, ciprofloxacin, and moxifloxacin in *Mycobacterium abscessus* complex subspecies as determined by phenotypic drug-susceptibility testing and GenoType NTM-DR assay^*a*^

	Number (%) of resistant isolates		
	*Mabs* (total *n* = 20)	*Mmas* (total *n* = 7)	*Mbol* (total *n* = 2)
pDST			
Azithromycin	13 (65.0%)	5 (71.4%)	2
Clarithromycin	12 (60.0%)	5 (71.4%)	2
Amikacin	3 (15.0%)	2 (28.6%)	1
Linezolid	3 (15.0%)	3 (42.9%)	–
Ciprofloxacin	8 (40.0%)	7 (100%)	2
Moxifloxacin	8 (40.0%)	7 (100%)	2
GenoType NTM-DR			
Inducible reistance/resistance to macrolides	14 (70.0%)	4 (57.1%)	2
Resistance to aminoglycosides	–	–	1

^*a*^ *Mabs*, *M. abscessus* subsp. a*bscessus*; *Mmas*, *M. abscessus* subsp. *massiliense*; and *Mbol*, *M. abscessus* subsp. *bolletti*; “-”, no resistant strain.

Amikacin resistance was detected in 20.7% (6/29) of isolates. The *rrs* gene mutation associated with amikacin resistance was confirmed in one isolate by GenoType NTM-DR and WGS. The diagnostic sensitivity of these methods was low (16.67%, 95% CI 0.42–64.12) but highly specific (100%, 95% CI 85.18–100.00).

Isolates resistant to other drugs were distributed as follows: 18 (62.1%) to moxifloxacin, 18 (62.1%) to ciprofloxacin, and 6 (20.7%) to linezolid.

A single mutation has been identified in MAB_4383c (I76V) protein (an efflux pump), and no mutations have been found in the MAB_4532c protein (*eis*2 gene).

Moreover, the follow-up pDST confirmed the evolve resistance to macrolides in 2/5 (40%) patients with serial isolates after 24 months of treatment.

### Transmission of MABC

**Fig 2:**
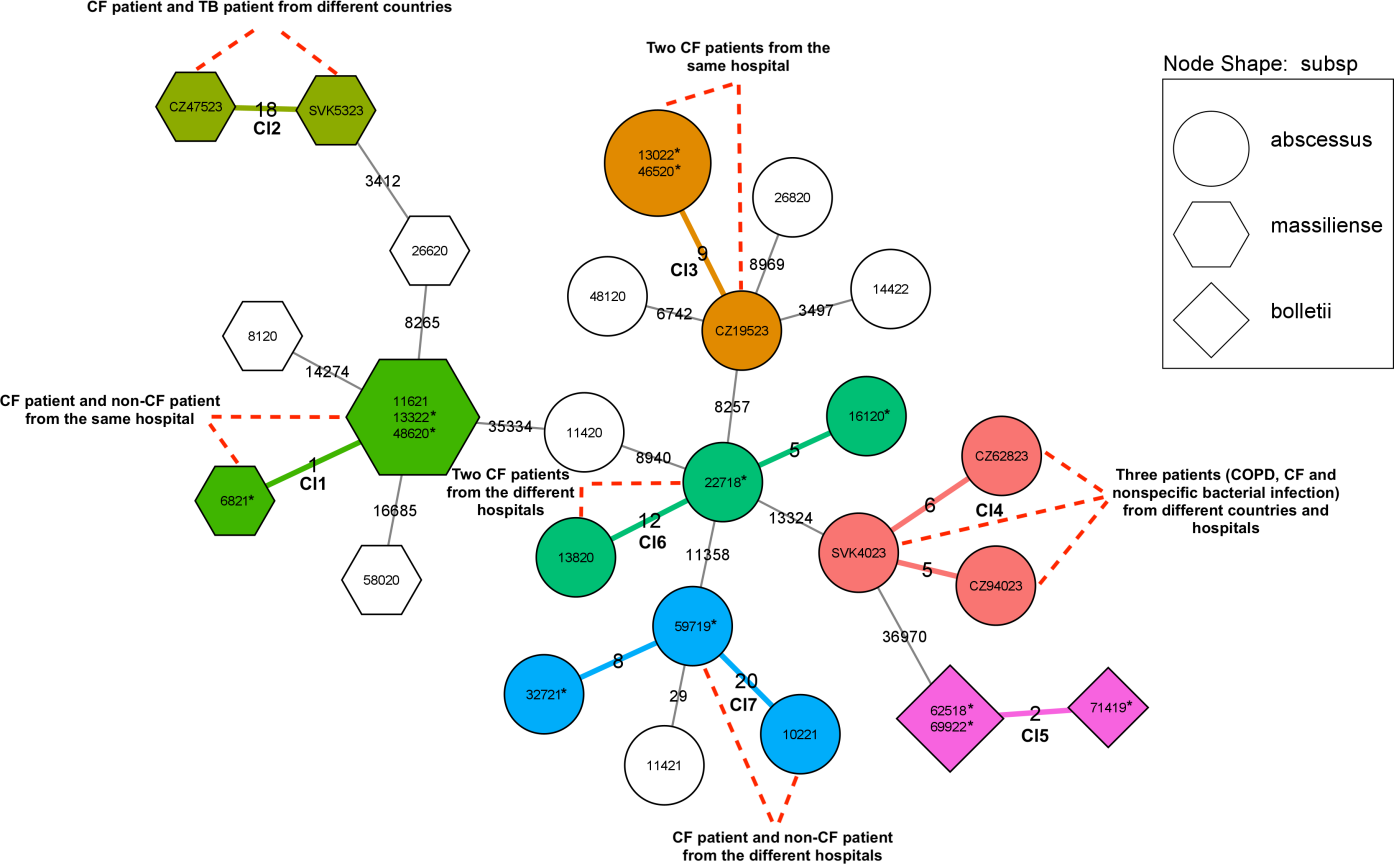
Minimum spanning tree representing the SNP differences among isolates from CF and non-CF patients within the *Mycobacterium abscessus* subspecies. Isolates belonging to the same cluster share the same color, while node shape differentiates between mycobacterial subspecies. The star symbol indicates serial isolates from the same patient. COPD, chronic obstructive pulmonary disease.

Using a threshold of 25 SNPs for closely genetically related isolates, the transmission analysis enables the identification of seven clusters (one formed by *Mbol*, two formed by *Mmas*, and four formed by *Mabs*) consisting of 21 isolates [from 14 patients (48.3%)], with a range of differences from 0 to 24 SNPs (Fig. 2). Five clusters included unique and serial isolates, while two were formed by serial isolates from the same patients (with a maximum of eight SNPs difference, Fig. 2). Serial isolates from the same patient were obtained for five individuals at least 12 months between sampling. Three of the intrapatient serial isolates were classified as *Mabs*, one *Mbol*, and one *Mmas*. The SNP differences between serial isolates ranged from 0 to 8, and there was no association between the number of SNPs and the time period between sampling. Our findings suggest that persistent infection (with partial microevolution and adaptation reflecting differences in SNPs) was a more significant factor than reinfection in these cases.

CF patients were likely to have a clustered isolate (71.4%). A single cluster comprised two isolates (*Mmas*) differing by 0 SNPs from two patients (one CF patient and one patient with malignant stomach cancer) admitted to the same hospital facility (Fig. 2, Cl1). According to epidemiological data, despite not being in the same ward, they may have undergone diverse examinations simultaneously, suggesting potential direct/indirect patient-to-patient transmission or exposure to a common environmental factor. Interestingly, clustered *Mmas* isolates exhibited variability in resistance to macrolides and linezolid.

Two clusters (Cl6, Cl7) comprised isolates (*Mabs*) that differed by 12 SNPs and 20 SNPs, respectively, obtained from CF patients in different centers (Fig. 2). Cl6 included patients younger than 18 years. Similarly, another cluster, Cl3, comprised two pediatric CF patients treated at the same center in different time periods (Fig. 2). Despite SNP differences inferring possible transmission events, epidemiological information does not reveal any direct contact. Although a direct route seems unlikely, gatherings of parents with children who have CF and similar events could be a contributing factor that should be considered. Therefore, we can presume that they acquired the infection from a common environmental source, or there may be other patients within the cluster have not been included in the study.

Additionally, we identified two international clusters involving isolates retrieved from two different countries (Slovakia and Czech Republic; Cl2, Cl4). In Cl2, *Mmas* isolates were obtained from patients diagnosed with CF and tuberculosis. Similarly, Cl4 comprised three closely genetically related isolates (<6 SNPs) recovered from patients with unspecified mycobacteriosis, chronic obstructive pulmonary disease, and CF. No direct epidemiological link has been confirmed between these patients.

### Classification of MABC isolates into DCCs

**Fig 3:**
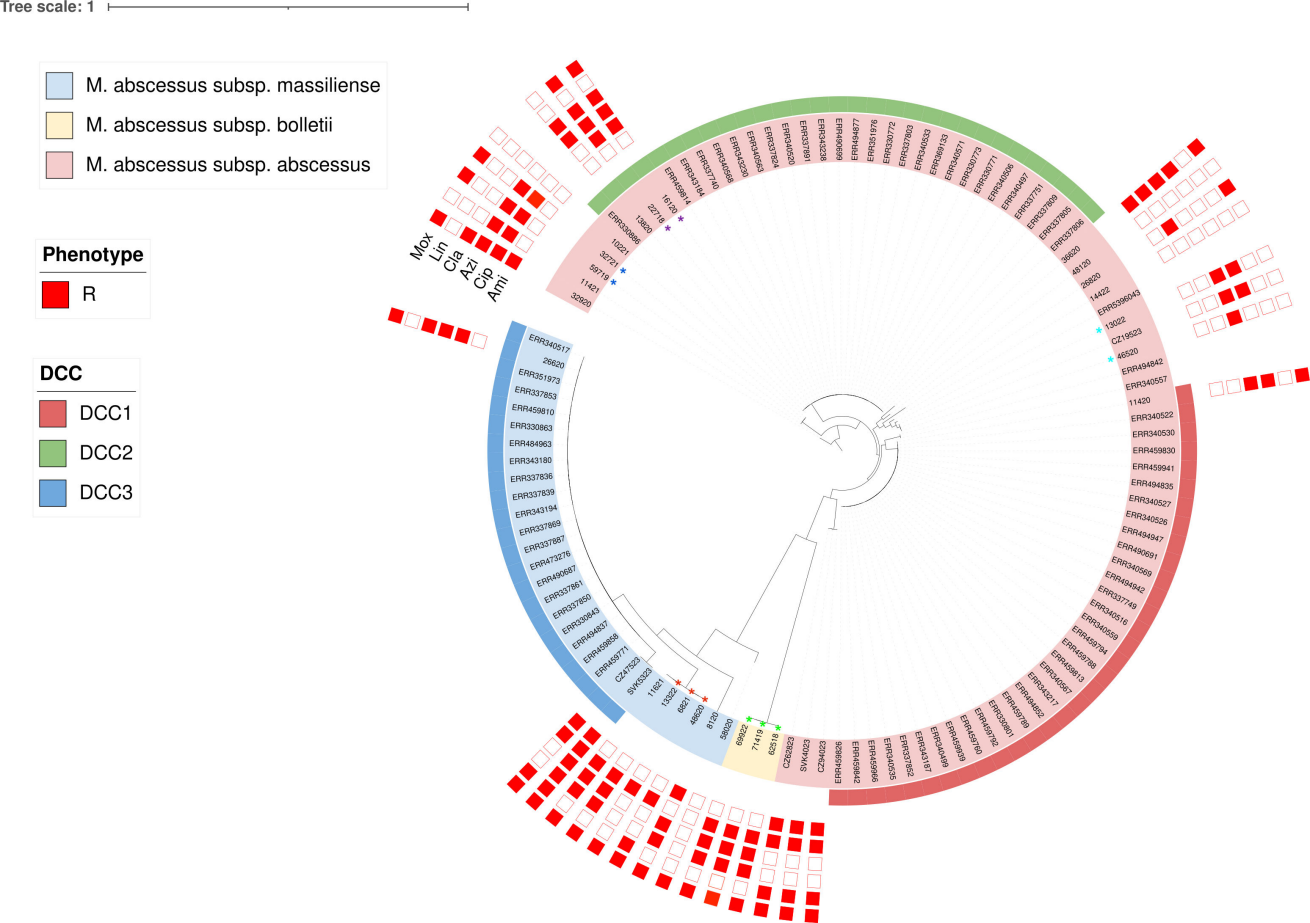
Phylogenetic tree comprising 110 *Mycobacterium abscessus* complex isolates illustrating integration of the isolates collected in the Czech Republic and Slovakia into specific DCCs. All isolates collected during our study are included along with associated resistance (red filled squares). Serial isolates from the same patient are denoted by a star symbol, with individual patients differentiated by color.

To analyze the associations between predominant *MABC* clones in the Czech Republic and previously described DCCs, publicly available sequencing data were employed. Within the strains analyzed in the study, seven (originating from six patients) were characterized to DCCs as follows: 114/20 (*Mabs*) belongs to DCC1; 161/20, 227/18, 138/20 (*Mabs*) belong to DCC2; 266/20, CZ47523, and SVK5323 (*Mmas*) belong to DCC3 (Fig. 3). Non-CF isolates were not distinct from CF isolates; instead, they were distributed across the phylogenetic tree, implying that specific clades of *MABC* do not show a predisposition for infecting CF patients.

### Genomic characterization of *Mmas* clustered isolates

**Fig 4:**
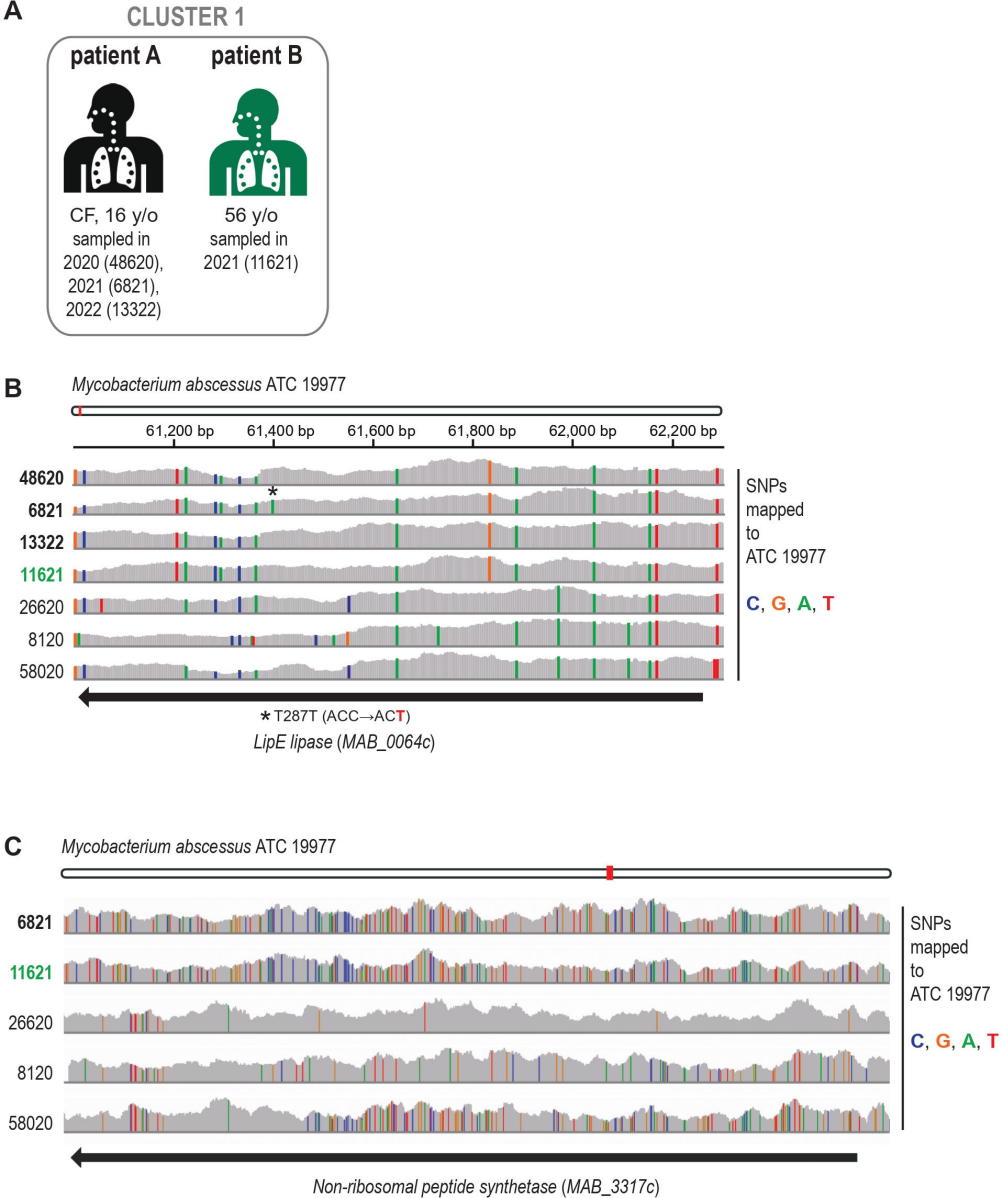
(A) "CF" indicates that the patient had cystic fibrosis. One patient was sampled three times, with sample number 48620 taken in 2020, sample number 6821 taken in 2021, and sample 13322 taken in 2022. Patients may have physically met during the year 2021 in the same hospital. (B) Comparison of *M. abscessus* subsp. *massiliense* isolates. Seven sequenced genomes of *M. abscessus* subsp. *massiliense* from patient isolates were mapped to the *M. abscessus* ATC 19977 reference genome. An ~1.2 kbp region is shown for illustration with positions of mutations in individual isolates when compared to the reference ATC 19977 genome. While three isolates from the Czech Republic vary among themselves, four genomes from isolates in Cluster 1 are almost identical. One of the very rare single nucleotide polymorphism is the synonymous mutation in LipE lipase, which was observed between three isolates from the same patient, samples 48620, 6821, and 13322. (C) The detected SNPs in ~8 kbp region encoding non-ribosomal peptide synthetase NRP (*MAB_3317c*). Sample numbers 6821 and 11621 represent isolated from the patients A and B, respectively, from Cluster 1. The *MAB_3317c* gene is highly variable among the *Mmas* isolates and belongs to the genes with the highest number of SNPs found in Cluster 1 samples, but not in the other *Mmas* isolates.

We performed a comparison between seven *Mmas* isolates with the reference *Mabs* ATCC 19977 genome to characterize unique mutations present exclusively in the genomes of nearly identical isolates within Cluster 1, originating from two patients hospitalized simultaneously at the same facility (Fig. 4A). These isolates are nearly identical; we detected one synonymous SNP in the MAB_0064c gene encoding LipE lipase (Fig. 4B). Notably, this SNP appeared in isolates from the same patient (patient A, Fig. 4A) sampled across different years. Rv3775 is a homolog of MAB_0064c and encodes LipE in *M. tuberculosis* H37Rv. Recently, Rv3775 was reported to be important for intracellular growth in macrophages and *in vivo* infection (22). However, the SNP we observed did not change the amino acid sequence.

Our analysis revealed approximately 1,200 mutations that are shared exclusively among all isolates within Cluster 1 (sample numbers 48620, 6821, 11621, and 13322) and absent in any of the other isolates (sample numbers 8120, 26620, and 58020, Table S2). Notably, around 350 of these mutations are nonsynonymous, affecting protein-coding genes. The highest number of nonsynonymous mutations was present in MAB_3317c gene encoding non-ribosomal peptide synthase. This gene was recently linked to *in vivo* survival, and its role in virulence was confirmed in Drosophila infection model (23). In addition, this gene was highly variable among *Mmas* isolates (Fig. 4C), indicating its potential contribution to the differences among *Mmas* strains.

## DISCUSSION

This is the first comprehensive study on molecular epidemiology and resistance profiling of *MABC* in the Czech Republic and Slovakia utilizing a cohort of clinical isolates obtained from CF and non-CF patients. Through analysis of related medical conditions and epidemiological data, we found a higher prevalence of MABC infection among CF patients under the age of 24. These results are supported by other studies showing that *M. abscessus* dominates in younger CF patients (including children), whereas the prevalence of infections caused by other NTM increases with age (24). The distribution of sublineages *Mabs* (69.0%), *Mmas* (24.13%), and *Mbol* (6.9%) is consistent with the findings of previous epidemiologic studies (25–27). Although the distribution of subspecies may vary among studies, such differences may reflect geographic locations and environmental source variations.

In the context of the results of the antimicrobial susceptibility tests, pDST confirmed the relatively high rate of isolates resistant to macrolides and fluoroquinolones. However, molecular testing demonstrated that up to 72.4% of *MABC* isolates harbored functional *erm*(41) or a mutation in the *rrl* gene. This is a concern because clarithromycin has demonstrated the strongest correlation between *in vivo* and *in vitro* sensitivity (28). Moreover, patients infected with macrolide-resistant *MABC* have demonstrated poorer clinical outcomes (7). Despite these findings, the prevalence of strains resistant to clarithromycin and ciprofloxacin remains similar or lower compared to recent studies (29–32). Phenotypic resistance to amikacin was relatively rare (six isolates, 20.7%), with only one isolate harboring a mutation in the *rrs* gene (based on WGS and Genotype test), indicating that resistance may be due to another mechanism. We investigated additional genomic regions involved in amikacin resistance and we could find a single mutation in MAB_4383c (I76V) protein, an efflux pump, which could be a plausible reason for *MABC* resistance (33). No mutations have been found on the *eis*2 gene (MAB_4532c protein), which has been recently proposed as a new enzyme to confer amikacin resistance in *MABC* (34). This result supports a recent study involving a global data set of *MABC* isolates, highlighting its potential as a first-line drug (29, 35, 36).

The studied strains showed relatively high genetic diversity, indicating numerous prevalent circulating strains in the countries (37). We confirmed the presence of seven clusters consisting of a total of 21 patients. The direct epidemiological link between the clustered patients was confirmed only in one cluster (Cl1), whereas the remaining clusters comprised closely related isolates obtained from various regions or even countries across, as shown in other studies (38, 39). Hence, we infer, consistent with the prior hypothesis, that strains of *MABC* may be spread asymptomatically by individuals lacking current coexisting conditions that elevate the likelihood of symptomatic infection, or through an unidentified environmental vector (39).

Considering the epidemiological link and the fact that three isolates in one cluster differed by 0 SNPs, we can assume that this is the supporting evidence of direct or indirect cross-transmission of *Mmas* among CF and non-CF patients as suggested in a recent study (14, 40, 41). This finding is noteworthy, given that past studies have identified clusters of both CF and non-CF patients, predominantly comprising non-CF patients who typically do not receive centralized medical care for their infections. Consequently, non-CF *Mmas* isolates are less likely to have epidemiological associations (42, 43). Moreover, isolates differing by 0 SNPs from patients with a proven epidemiological connection are rare, as usually the direct epidemiological link is absent in the clustered patients (44). Recently, near-identical *Mmas* isolates, generally differing by fewer than 22 SNPs, were found among non-CF patients with progressive neurodegenerative diseases (41). However, these patients were long-term hospitalized, often in the same wards, and ventilator dependent. Nevertheless, *Mmas* was isolated from one air sample obtained near a positive patient, suggesting airborne transmission.

In order to describe potential mutations that play a key role in patient-to-patient transmission, we analyzed the mutations shared exclusively among all *Mmas* isolates within Cl1 and absent in any of the other isolates and found the highest number of nonsynonymous mutations present in a gene encoding non-ribosomal peptide synthase (*MAB_3317*c) known to be associated with *Mabs* virulence (23).However, further comparison with other *Mmas* isolates suspected of possible patient-to-patient transmission will be necessary to determine if this gene (probably together with other genes) could be associated with a higher transmission probability. These data will become more available in the future as more isolates become whole-genome sequenced.

In accordance with prior studies, we found clustered isolates (pairwise SNP distance of less than 20) from patients with both CF and non-CF hospitalized in the same setting at different times or even from diverse geographic regions/countries (39, 43). There were no obvious epidemiological links between patients in these clusters; therefore, we assumed that recent transmission would not explain close relationships between these patients, rather we suggest the point source or independent acquisition from environmental sources. In the future, it will be interesting to compare all strains reported to have the potential for cross-transmission, especially as more sequenced genomes become available. Nevertheless, identifying genetically linked *MABC* strains in transmission analysis requires careful interpretation concerning global molecular epidemiology (24).

Furthermore, the maximum SNP distance within paired isolates was 8, consistent with a previous report (45). Also, we found that the majority of patients hospitalized in the same facility in this study had unique strains. Thus, if there were frequent instances of direct patient-to-patient transmission, we would expect to observe evidence of it in this context.

Regarding phylogenetic analysis, we classified seven isolates into three different DCCs. All of these isolates exhibited resistance to macrolides and, except one, to fluoroquinolones. While DCC isolates are recognized for their greater resistance, higher virulence, and worse clinical outcomes, our data set shows that most isolates, regardless of the DCC, indicate the possible emergence of multidrug-resistant strains (12, 17). The isolates classified as DCC in our analysis were all but one restricted to patients with CF despite findings in other studies indicating that DCC can also infect individuals without CF (15, 16). One international cluster containing two *Mmas* isolates belonging to DCC3 was identified. However, no apparent sources of infection were identified through the available epidemiological links of these patients. Similar findings were observed in several recent studies, implying that transmission chains might involve unidentified links, possibly implicating environmental, cross-border transmission, or human intermediaries (27, 44).

Our study had some limitations. We could not access comprehensive epidemiological meta-data for all clustered patients and those assigned to DCCs. This includes information on whether patients had interactions outside the hospital, such as private meetings or CF patient gatherings, and whether they had a history of hospitalization outside the Czech Republic. Additionally, we did not collect isolates from healthcare settings; thus, we could not compare the genetic similarity between respiratory and healthcare environmental isolates. However, other studies suggest that healthcare-associated transmission of *MABC* is rare, as this bacterium is rarely isolated from the closest environment or lacks genetic similarity to the patient’s isolate (6, 41).

To conclude, we have confirmed the high rate of drug resistance to commonly used antibiotics within the *MABC* subspecies with the lowest level of resistance to amikacin and characterized the population most affected by *MABC* infections. Additionally, the WGS demonstrated a robust discriminatory capacity for analyzing *MABC* strains prevalent in the Czech Republic. It enabled the identification of clusters involving patients infected or colonized with closely related isolates (with less than a 25 SNP distance), indicating the occurrence of genetically similar strains across different geographical settings. This funding emphasizes the application of WGS and resistance profiling in the MABC surveillance system. Also, it will assist public health authorities in managing infection prevention and potentially preventing the onward transmission of *MABC*.

## Data Availability

Sequence data relating to all isolates have been deposited in the Sequence Read Archive under BioProject number PRJNA1051626.

## SUPPLEMENTAL MATERIAL

Table S1 spectrum.01056-24-s0001.xlsx Summary of resistance profiles determined by molecular and phenotypic methods.

Table S2 spectrum.01056-24-s0002.xlsx List of mutations identified in Cluster 1 isolates.

ASM does not own the copyrights to Supplemental Material that may be linked to, or accessed through, an article. The authors have granted ASM a non-exclusive, world-wide license to publish the Supplemental Material files. Please contact the corresponding author directly for reuse.
